# Genome-wide variation landscape reveals temperature adaptation in Chinese indigenous cattle

**DOI:** 10.1186/s40104-026-01451-6

**Published:** 2026-07-01

**Authors:** Changheng Zhao, Jun Teng, Yan Chen, Cheng Yang, Xinyi Zhang, Chao Ning, Huili Wang, Qien Yang, Wenfa Lv, Dan Wang, Qin Zhang

**Affiliations:** 1 Shandong Provincial Key Laboratory for Livestock Germplasm Innovation & Utilization, College of Animal Science and Technology, Shandong Agricultural University, Tai’an, 271018 China; 2 College of Animal Science and Technology, Hebei Agricultural University, Baoding, Hebei 071001 China; 3 Institute of Animal Science, Jiangsu Academy of Agricultural Sciences, Nanjing, 210014 China; 4 CAS Key Laboratory of Adaptation and Evolution of Plateau Biota, Qinghai Key Laboratory of Animal Ecological Genomics, Northwest Institute of Plateau Biology, Chinese Academy of Sciences, Xining, Qinghai 810001 China; 5 Key Lab of Animal Production, Product Quality and Security, Ministry of Education, Jilin Agricultural University, Changchun, 130118 China

**Keywords:** Chinese indigenous cattle, Genome-wide variation, Population structure, Selective sweeps, Temperature adaptability

## Abstract

**Background:**

The significant temperature variations across northern and southern China have driven the adaptive evolution of Chinese native cattle breeds, allowing them to thrive in diverse and extreme bioclimate environments. Understanding how these breeds have adapted to varying temperatures is essential for identifying genetic factors that contribute to their survival in such conditions.

**Results:**

In this study, using whole-genome sequence data of 336 individuals (with an average sequencing depth of 30.12 ×) from 21 cattle breeds, including 8 breeds from cold regions, 3 from warm regions, and 10 from hot regions, clear genetic differentiation among the three groups of breeds was revealed. Using whole-genome SNP, InDel, and SV data, a series of selective genomic regions, genes, and variants/SVs associated with cold or hot temperature adaptability were identified. Key genes, including *KLB*,* HSPA4*,* ECSCR*,* DNAJC18* and *SLC9A1* are speculated to be responsible for cold/hot adaptability based on the extreme difference in allele frequency of the selective variants/SVs harbored by these genes, their known biological functions, protein–protein interaction network, findings from previous studies on their relation to environmental adaptation, and their tissue specificities.

**Conclusions:**

By integrating SNP, InDel, and SV data, this study provides a comprehensive genetic framework for understanding selective environmental adaptation. These findings enhance our understanding of the mechanisms underlying temperature adaptation in cattle and offer a molecular foundation for the development of new breeds.

**Supplementary Information:**

The online version contains supplementary material available at 10.1186/s40104-026-01451-6.

## Background

As one of the key ruminant species, cattle provide a wide range of human needs by supplying leather, meat, and milk, while also serving as draft animals for tasks like cart-pulling and plowing, especially in less industrialized regions [1]. Domestic cattle are divided into humpless taurine (*Bos taurus taurus*) and humped indicine (*Bos taurus indicus*) [2]. Taurine cattle are predominantly found in regions with temperate to cold climates, characterized by their smooth, streamlined body and tight skin. In contrast, indicine cattle, mostly living in subtropical and tropical regions with hot and/or humid climates, are distinguished by several unique morphological and physiological traits, including a prominent muscular hump over the shoulders, a well-developed dewlap, and drooping ears. Additionally, indicine cattle exhibit a lower basal metabolic rate, reduced water and nutrient requirements, and greater resistance to tick infestations and gastrointestinal parasites compared to taurine cattle [3, 4].

China, located in eastern Asia along the western Pacific coast, spans both the eastern and northern hemispheres, encompassing diverse geographical, climatic, and ecological conditions. The significant temperature variations from north (cold) to south (hot) have driven the evolutionary adaptation of native cattle breeds, allowing them to thrive in diverse and extreme temperatures. Chinese indigenous cattle are classified into three groups based on their geographical distribution and genomic lineage: (a) southern breeds, primarily derived from the indicine lineage; (b) northern breeds, belonging to the taurine lineage; and (c) central breeds, which are hybrids of taurine and indicine cattle [5, 6]. Investigating climate-driven selection signatures is crucial for understanding the genetic basis of environmental adaptability and identifying the corresponding functionally genetic variants.

In recent years, whole genome sequencing (WGS) has been widely used to identify genetic variations that are influenced by environmental adaptation in domesticated livestock, including pigs, chickens, sheep, goats, cattle, and horses [7–12]. Several studies investigated the temperature adaptability of cattle. For instance, Ghoreishifar et al. [13] examined selective regions associated with the adaptation of cattle to cold climates in northern Sweden (Fjäll or Swedish mountain cattle). Li et al. [14] explored the selection signatures of Dehong humped cattle, a breed renowned for its adaptation to tropical climates, to gain deeper insights into the biological mechanisms underlying hot tolerance. Despite these numerous efforts, our understanding of the genetic basis of the temperature adaptability of cattle remains limited.

Up to now, selective sweep analyses primarily rely on SNP markers, while WGS offers the opportunity to capture a broader spectrum of variants across the genome, including insertions/deletions (InDels) and structural variations (SVs). InDels are the second most common type of genomic variants [15], and have been shown in human studies to play crucial roles in evolutionary changes [16, 17], and also extensively used in livestock population genetic analyses [18–21]. SVs have significant functional impacts on genomic evolution and local adaptation in humans [22, 23] and livestock [24–27]. On a molecular scale, they can impact gene dosage, gene expression, DNA interactions, and the three-dimensional structure of the genome by modifying the proximity and copy number of genetic elements [28, 29]. Some previous studies investigated the distribution of SVs in the genomes of cattle [30, 31] and found significant associations between SVs and some phenotypes [32, 33]. However, research on the association of InDels and SVs with temperature adaptability is still rare.

In this study, we analyzed the whole genome sequence data of 336 cattle with an average sequencing depth of 30.12 ×, representing 21 cattle breeds, including 8 breeds from cold environments (6 northern Chinese indigenous breeds and 2 foreign taurine breeds), 3 breeds from warm environments (3 central Chinese indigenous breeds), and 10 breeds native to hot environments (7 southern Chinese indigenous breeds and 3 foreign indicine breeds). We investigated the population structures of these breeds and identified selective genomic regions and genetic variations (SNPs, InDels and SVs) associated with adaptation to cold or hot environments. Our findings offer new insights into population stratification and temperature-induced adaptive selection signatures in the cattle genome.

## Methods

### Data information

Blood samples were collected and sequenced from 235 cattle of eight Chinese indigenous breeds: Altay White-Headed, Fuzhou, and Yanbian from the northern region; Wenling Humped and Zhoushan from the southern region; and Luxi, Mengshan, and Xuzhou from the central region. In total, 23,784 Gb of sequence data were generated. Additionally, sequence data for 101 cattle were obtained from the National Center for Biotechnology Information (NCBI) database, representing 10 Chinese indigenous breeds (with Yanbian and Zhoushan overlapping with the breeds sequenced in this study) and five foreign breeds. The average sequencing depth for the 336 individuals was 30.12 ×, ranging from 10.28 × to 78.36 ×. The annual mean temperature and the lowest temperature of the regions where the 21 breeds are located were obtained from WorldClim version 2 (https://www.worldclim.org) and Weather and Climate (https://weatherandclimate.com). The detailed information about these breeds, along with their corresponding sequencing data and climatic variables, is provided in Table 1 and Additional file 1: Table S1. In brief, the numbers and breeds/individuals in the cold, hot, and warm groups were 8/122, 10/117, and 3/97, respectively. The average and minimum temperatures were −9.39 °C to 9.64 °C and −25.0 °C to −53.8 °C for the eight cold group breeds, 18.45 °C to 30.26 °C and 2.60 °C to 12.90 °C for the 10 hot group breeds, and 12.96 °C to 14.45 °C and −9.0 °C to −12.0 °C for the three warm group breeds.

**Table 1:** Twenty-one cattle breeds involved in this study and their average sequencing depths (± SD), numbers of animals, and bioclimate variables (annual mean temperature and minimum temperature) of their distribution regions

Breed	Code	Land of origin	Number	Group	Depth	Mean, °C	Minimum, °C
Altay White-Headed	ALT	Altay, Xinjiang, China, Asia	30	Cold	40.91 ± 6.36	4.45	−41.00
Finncattle	FIN	Finland, Europe	8	Cold	12.67 ± 0.24	5.64	−31.45
Fuzhou	FZ	Wafangdian, Liaoning, China, Asia	28	Cold	16.19 ± 7.45	9.64	−25.00
Hasake	HSK	Yili, Xinjiang, China, Asia	9	Cold	13.06 ± 1.30	7.66	−26.00
Qaidam	QDM	Haixi, Qinghai, China, Asia	8	Cold	25.69 ± 17.61	3.97	−25.00
Tibetan	TBT	Changdu, Xizang, China, Asia	8	Cold	13.96 ± 2.39	2.90	−29.00
Yakut	YKT	Yakutia, Russia, Asia	5	Cold	12.41 ± 0.35	−9.39	−53.80
Yanbian	YB	Yanbian, Jilin, China, Asia	26	Cold	34.88 ± 12.29	5.53	−28.00
Luxi	LX	Heze, Shandong, China, Asia	41	Warm	39.40 ± 7.01	14.00	−9.00
Mengshan	MS	Linyi, Shandong, China, Asia	42	Warm	40.40 ± 9.27	12.96	−10.00
Xuzhou	XZ	Xuzhou, Jiangsu, China, Asia	14	Warm	37.07 ± 3.69	14.45	−12.00
Brahman	BRM	Brahman, West Bengal, India, Asia	4	Hot	17.10 ± 2.16	30.26	11.00
Dianzhong	DZ	Puer, Yunnan, China, Asia	8	Hot	11.15 ± 0.86	18.92	6.30
Guangfeng	GF	Shangrao, Jiangxi, China, Asia	4	Hot	11.93 ± 1.20	18.53	2.60
Ji’an	JA	Ji’an, Jiangxi, China, Asia	4	Hot	12.51 ± 0.83	18.61	3.30
Kenana	KNN	Sudan, Africa	9	Hot	11.07 ± 0.18	30.04	10.13
Leiqiong	LQ	Leizhou, Guangdong, China, Asia	9	Hot	26.83 ± 10.28	23.51	12.90
Ogaden	OGD	Ethiopia, Africa	9	Hot	10.87 ± 0.45	22.23	6.82
Wenling humped	WL	Wenling, Zhejiang, China, Asia	30	Hot	39.18 ± 5.61	19.09	3.80
Wenshan	WS	Guangnan, Wenshan, China, Asia	7	Hot	13.54 ± 2.23	19.24	5.80
Zhoushan	ZS	Zhoushan, Zhejiang, China, Asia	33	Hot	34.50 ± 12.25	18.45	2.60
Total			336		30.12 ± 14.02		

### SNPs/InDels calling

The raw sequencing reads were filtered using Fastp v0.23.2 [34] to obtain clean reads. SNPs and InDels were called using the Sentieon DNASeq pipeline (https://www.sentieon.com/products/). The process involved mapping the cleaned reads to the cattle reference genome ARS-UCD1.2 using the “bwa mem” function, followed by sorting the BAM files and removing duplicates with the “util sort” and “Dedup” functions. Raw GVCFs were generated from the bam files using the “Haplotyper” function, and the individual GVCFs were merged and jointly called to generate VCFs using the “GVCFtyper” function. To avoid potential false-positive calls, the “VariantFiltration” function of GATK v4.2.6.1 [35] was applied to filter SNPs with the following criteria: QD (quality by depth) < 2.0, MQ (mapping quality) < 40.0, FS (Fisher strand) > 60.0, SOR (strand odds ratio) > 3.0, MQRankSum (mapping quality rank sum test) < −12.5, and ReadPosRankSum (read position rank sum test) < −8.0. For InDel filtering, the criteria were: QD < 2.0, FS > 200.0, SOR > 10.0, MQRankSum < −12.5, and ReadPosRankSum < −8.0. Non-biallelic SNPs/InDels and SNPs located within 5 base pairs (bp) of InDels were then removed, resulting in a total of 81,759,453 SNPs (on average one per 30 bp), and 8,925,961 InDels (length ≤ 50 bp, on average one per 279 bp) on the autosomes (Additional file 1: Table S2).

SNPs/InDels with a minor allele frequency (MAF) ≤ 0.05 and a missing rate > 10% were excluded, retaining a total of 26,077,580 SNPs and 2,519,603 InDels for subsequent analysis.

### Structural variants calling

In light of the comprehensive benchmarking by Cameron et al. [36], which showed that Lumpy achieves higher sensitivity than other short-read SV callers, we performed SV discovery with Lumpy v0.3.1 [37] on aligned BAM files to detect deletions (DEL), duplications (DUP), and inversions (INV). Per-sample calls were merged across all individuals using SVtools v0.4.0 [38], genotyped with SVtyper v0.7.0 [39], and annotated for read-depth with Duphold v0.2.1 [40]. Following the recommendations of Wold et al. [41] and Pedersen and Quinlan [40], raw callsets were filtered on call quality and genotype quality. Duphold flank fold-change (DHFFC) thresholds were applied by only retaining putative DELs with “DHFFC < 0.7” and DUPs with “DHFFC > 1.3”. All SVs were further filtered by the Mean Smoove Heterozygote Quality (MSHQ) score with BCFtools v1.15.1 [42], retaining only SVs with an MSHQ score ≥ 3. Finally, SVs shorter than 50 bp were removed, yielding 112,233 autosomal SVs (on average one per 22,181 bp), comprising 103,255 DELs, 7,795 DUPs, and 1,183 INVs (Additional file 1: Table S2).

SVs with MAF ≤ 0.05 and missing rate > 10% were filtered out, and 24,707 high-quality SVs were retained for subsequent analyses.

### Population structure and genetic diversity analysis

Genetic distances among all individuals were calculated using the VCF2Dis software [43] and SNP markers, and the results were input into FastMe2.0 [44] to generate the Neighbor-Joining (NJ) phylogenetic tree, with yak as the outgroup. The resulting phylogenetic tree was visualized using the iTOL program [45]. A population-level phylogeny was also inferred using the maximum likelihood approach implemented in TreeMix [46]. Principal component analysis (PCA) was performed using PLINK v1.9 [47]. For admixture analyses, linkage disequilibrium (LD)-based pruning was first performed on the genotype data with the threshold of *r*^2^ ≥ 0.2 within a 50-kb window using PLINK v1.9. ADMIXTURE v1.3.0 [48] was then applied to quantify genome-wide admixture across these breeds, with the putative number of populations (*K* values) ranging from 2 to 10.

The genetic distances, measured with fixation statistic (*F*_*ST*_) [49], between the 21 cattle breeds were calculated using Python scripts (https://github.com/simonhmartin/genomics_general/popgeneWindows.py). The nucleotide diversity (π) [50] for each breed was calculated using VCFtools v0.1.13 [51].

### Selective sweep detection

Selective sweeps in the genome with respect to temperature adaptability in the cold and hot breed groups were identified using SNPs, InDels, and SVs, respectively. For the detection of selective sweeps in the cold group, the hot group was used as the control group. Conversely, for the detection of selective sweeps in the hot group, the cold group was employed as the control group. When using SNPs/InDels, four statistics, including *F*_*ST*_ [49], θπ ratio (θπ-control/θπ-target) [50], XPCLR (cross-population composite likelihood ratio) [52], and XP-EHH (cross-extended haplotype homozygosity) [53], were combined into a single statistic, i.e., de-correlated composite of multiple signals (DCMS), as described in Ma et al. [54]. These statistics were calculated for each 50-kb sliding window with a 20-kb step. The *F*_*ST*_ and θπ values were calculated using VCFtools v0.1.13 [51], and the θπ ratios were log2-transformed (log_2_(θπ ratios)). The XPCLR values were calculated using XP-CLR v1.0 [52]. The XP-EHH values were calculated for each SNP/InDel using the SELSCAN V1.1 software [55], and then averaged for each window. The DCMS combines the *P*-values of the four statistics for each window into a single measure considering the correlations between them. The genome-wide correlation matrix was calculated, and different weights were given to the *P*-values of the four statistics according to their genome-wide correlations. Following recommendations from the previous studies [56, 57], DCMS with a *P*-value < 0.001 were regarded as significant, and windows with significant DCMS were considered as selective regions. Overlapped selective regions were merged.

When using SVs, given the sparse density of SVs, the method θπ ratio, XPCLR, and XP-EHH are not applicable. The θπ ratio is inherently SNP/InDel-based (nucleotide diversity), whereas XPCLR and XP-EHH require dense, accurately phased SNP/InDel haplotypes. Applying these statistics directly to SVs would introduce bias and impair comparability. SV-based selective sweeps were therefore scanned exclusively with* F*_*ST*_, which depends only on between-population allele frequencies and has been widely used to quantify SV-based population differentiation [26, 58, 59]. Specifically, differentiation between the cold and hot groups was evaluated using *F*_*ST*_, and SVs with a *P*-value < 0.001 were designated as candidate selective SVs [60].

### Gene enrichment analysis and protein–protein interaction network

Gene annotation was performed using the *Bos taurus* annotation file (https://ftp.ncbi.nlm.nih.gov/genomes/all/GCF/002/263/795/GCF_002263795.1_ARS-UCD1.2/GCF_002263795.1_ARS-UCD1.2_genomic.gtf.gz) to identify genes within or overlapping with the selective regions/SVs. Gene set enrichment analysis was conducted using OmicShare tools (http://www.omicshare.com/tools) to identify Kyoto Encyclopedia of Genes and Genomes (KEGG) pathways, with *P* < 0.05 considered significantly enriched. The protein–protein interaction (PPI) network was constructed based on the STRING database [61] (minimum interaction score ≥ 0.400, medium confidence) and visualized using Cytoscape v3.10.4 [62]. The top 5 genes with the strongest connectivity in the network were selected as hub genes.

### Variant annotation and identification of selective variants

The variants (SNPs, InDels, and SVs) involved in the selective regions were annotated using SnpEff v5.2 [63] with the genome annotation file mentioned above to predict their consequence impact on protein sequence, which are classified as high (splice acceptor, splice donor, stop gained, frameshift, stop lost and start lost), moderate (missense, in-frame insertion, and in-frame deletion), low (synonymous), and modifier (intron, upstream/downstream gene, UTR or intergenic variants). Variants with high-impact consequences and missense SNPs were selected for further analysis. Allele frequencies for the selected variants in the hot, warm, and cold groups were calculated, and variants with a large absolute allele frequency difference (ΔAF > 0.75) between the hot and cold groups were considered to be selective. In addition, for missense SNPs, the local structural characteristics of the wild type and mutant type proteins were assessed using DynaMut2 [64] to predict the thermodynamic stability (ability to keep its original structure and function) changes before and after the mutation.

### Validation of selective variants

To further explore the selective variants (SNPs and InDels) identified above, genotype data for 119 cattle of seven cattle breeds adapted to cold environments were extracted from the 1000 Bull Genomes Project (Run8), including 20 Buryat, 19 Finncattle, 11 Fjäll, 4 Kazakh White-Headed, 20 Kholmogory, 35 Yakut, and 10 Yaroslavl cattle. The allele frequencies of these variants in these 119 cattle and in the cold group of this study were then compared.

### Tissue-specificity analysis of selective genes

From the Cattle Genotype-Tissue Expression atlas (CattleGTEx) [65] datasets, 23 tissues with a sample size greater than 50 were selected to analyze the tissue-specificities of the selective genes (genes located within or overlapping with the identified selective regions/SVs). The transcripts per million (TPM) values for these genes across the 23 tissues were obtained, and their tissue specificity indexes (*tau*) [66], ranging from 0 for housekeeping genes to 1 for fully tissue-specific genes, were calculated using the tspex v0.6.1 software [67]. Following the recommendation of Kryuchkova-Mostacci and Robinson-Rechavi [68], genes with a *tau* value ≥ 0.80 were considered to have high tissue specificity.

## Results

### Population structures and genomic diversities

**Fig. 1:**
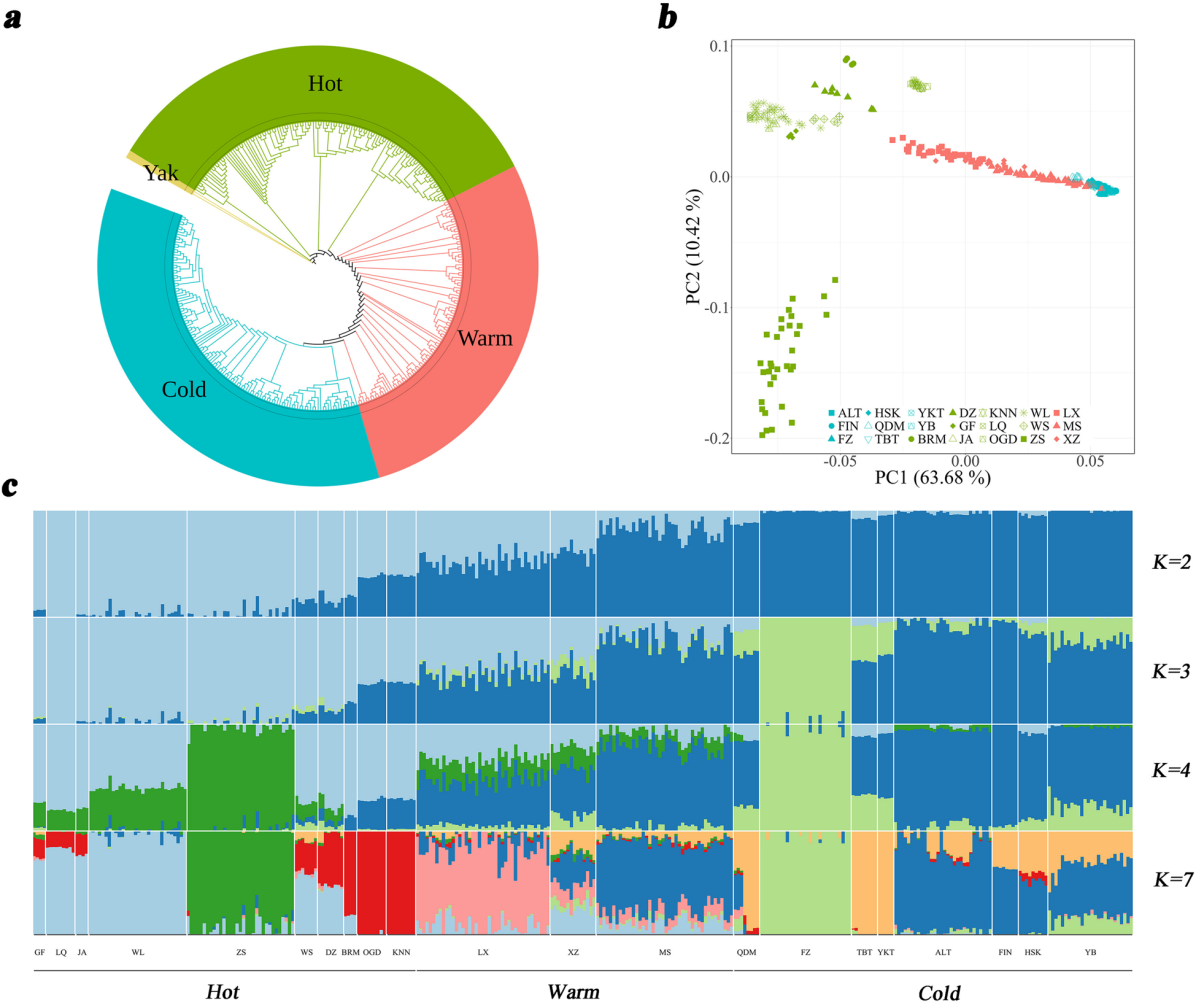
Population structure analysis of 21 cattle breeds involved in this study. **a** The neighbor-joining tree of the 21 breeds constructed using whole autosomal sequence SNP data, with yak as the outgroup. **b** Results of principal component analysis (PC_1_ versus PC_2_) of the 21 breeds. **c** Results of admixture analysis of the 21 breeds (*K* = 2, 3, 4, 7)

In the phylogenetic tree (Fig. 1a), the 21 breeds were clearly clustered into three groups, which is fully consistent with the grouping based on the temperature of their geographical locations. The same differential topology was observed in the maximum likelihood tree of these breeds (Additional file 2: Fig. S1). The principal component analysis revealed clear phylogeographical differentiation (Fig. 1b), with PC1 showing differentiation between indicine and taurine cattle and PC2 further separating ZS from the hot group. The admixture analysis recapitulated a similar pattern (Fig. 1c). At *K* = 2, the populations were basically classified into two groups: taurine cattle and indicine cattle. The taurine cattle consisted predominantly of the breeds of the cold group, while the indicine cattle consisted predominantly of the breeds of the hot group. The warm group displayed an admixture of taurine and indicine lineages, with LX exhibiting the highest proportion of indicine ancestry. At *K* = 3, FZ was clearly separated from the other breeds, while at *K* = 4, ZS was distinctly isolated from the remaining breeds. The analysis reached the lowest cross-validation error when *K* was increased to 7.

**Fig. 2:**
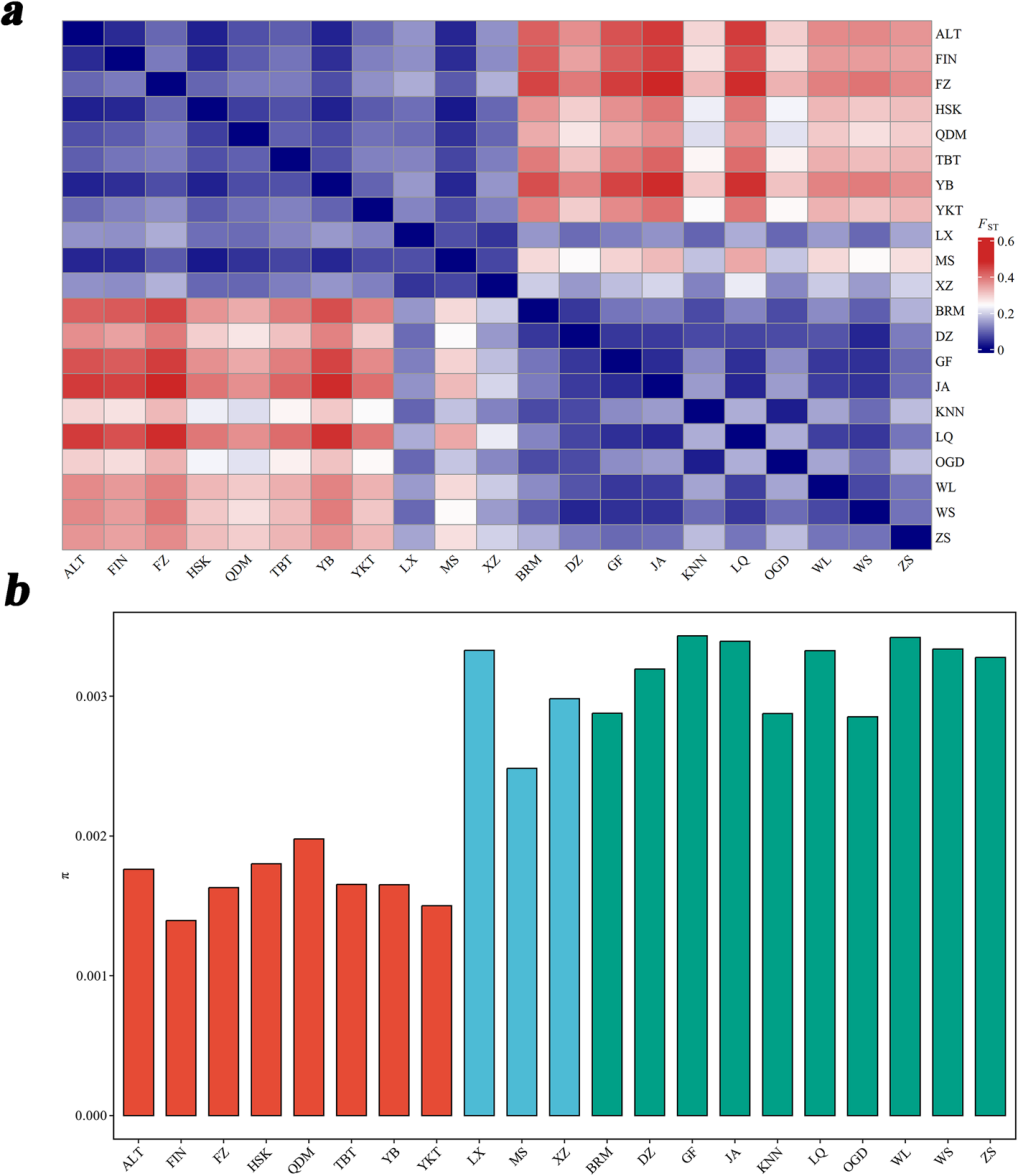
Distance matrix (in terms of *F*_*ST*_) and nucleotide diversity (π) of the 21 cattle breeds. **a** Distance matrix (in terms of *F*_*ST*_) among the 21 cattle breeds. **b** Nucleotide diversities (π) of the 21 cattle breeds

The genetic distances among the 21 breeds are given in Fig. 2a and Additional file 1: Table S3. The within-group genetic distances were significantly smaller than the between-group distances in all three groups. Specifically, the *F*_*ST*_ values ranged from 0.03 (among ALT, HSK, and YB) to 0.14 (between YKT and FZ) within the cold group, from 0.05 (between LX and XZ) to 0.08 (between LX and MS) within the warm group, and from 0.03 (between KNN and OGD) to 0.18 (between ZS and KNN/OGD) within the hot group. For between-groups, the *F*_*ST*_ values ranged from 0.21 (between KNN and QDM) to 0.49 (between FZ and JA).

The nucleotide diversities measured with π values for each breed are shown in Fig. 2b and Additional file 1: Table S4. The breeds in the cold group had the lowest π values, ranging from 0.0014 (for FIN) to 0.0020 (for QDM). The breeds in the warm group exhibited π values ranging from 0.0025 (for MS) to 0.0033 (for LX). For the hot group, the π values for the three foreign breeds were all 0.0029, while those for the seven southern Chinese indigenous cattle breeds were 0.0032–0.0034.

### Selective regions and variants identified in the cold group using SNPs and InDels

**Fig. 3:**
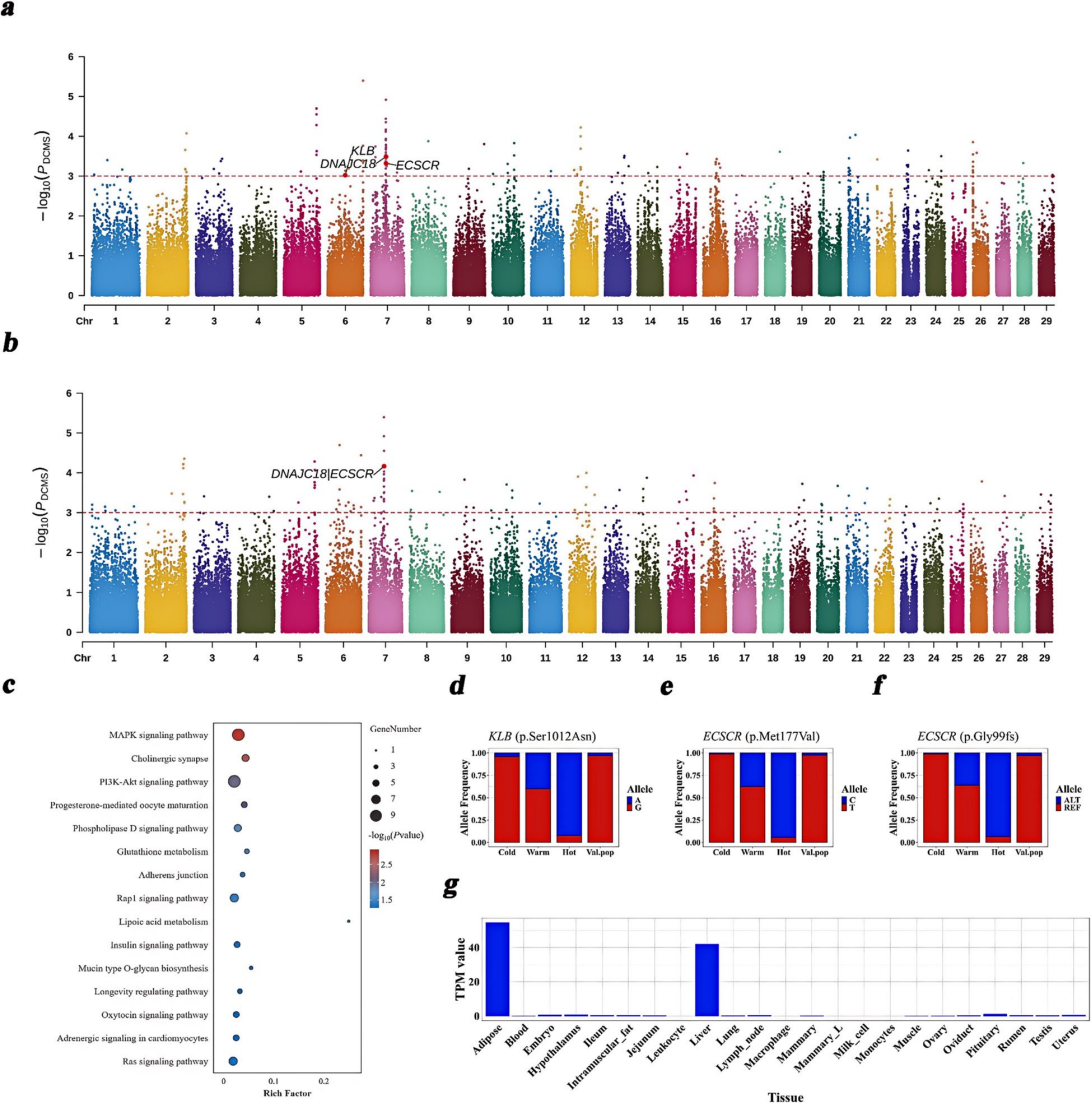
Identification of selective sweeps in the cold group based on SNP and InDel data. **a** and **b** Manhattan plots of the *P*-values (−log_10_(*P*)) (*y*-axis) in sliding 20 kb-windows across all autosomes (*x*-axis) based on SNP data and InDel data. The red dashed line indicates the significance threshold (*P*-value = 0.001). **c** The Sankey-Dot plot of the top 15 KEGG pathways of the selective genes (genes involved in the significant selective regions). **d**–**f** The gene frequencies of the selective variants (missense SNPs and frameshift InDel) in the *KLB* and *ECSCR* genes in the cold, warm, and hot groups and in the validation population. **g** TPM (Transcripts per million) values of the *KLB* gene across 23 tissues

Figure 3a and b illustrate the genomic selective regions identified in the cold group using the SNP and InDel data, respectively, with the hot group as the control. The correlation between the *P*-values derived from the SNP and InDel windows was calculated, yielding a correlation coefficient of 0.57. Using the SNP data, 86 selective regions encompassing 160 genes were identified (Additional file 1: Table S5), while 103 selective regions covering 149 genes were identified based on the InDel data (Additional file 1: Table S6). Notably, 15 selective regions and 24 genes identified from InDel data overlapped with those identified from SNP data.

To explore the biological significance of the identified genes, KEGG analysis was performed for all 285 genes involved in the selective regions. This analysis revealed 14 significant pathways (Fig. 3c and Additional file 1: Table S7), which were mainly related to signal transduction (MAPK signaling pathway; PI3K-Akt signaling pathway, Phospholipase D signaling pathway), nervous system (Cholinergic synapse), metabolism (Glutathione metabolism; Lipoic acid metabolism), and endocrine system (Insulin signaling pathway). The MAPK (mitogen-activated protein kinase) signaling pathway is generally considered a survival pathway that enhances cold resistance in animals [69, 70]. The cholinergic synapse signaling pathway is essential for multiple neurodevelopmental processes, including axon regeneration and guidance [71, 72]. The insulin signaling pathway, which regulates lipid and carbohydrate metabolism and is highly conserved across species from insects to mammals [73, 74], is suggested to be influenced by temperature [75, 76].

PPI analysis was performed on the 285 genes using the STRING database. After excluding unconnected nodes, a PPI network comprising 91 genes was constructed (Additional file 3: Fig. S2). Among them, the five genes, *DNAJC18*, *SIL1*, *SPATA24*, *ECSCR*, and *SMIM33,* with the strongest connectivity were identified as hub genes.

To further understand the genetic basis of cold adaptation, the variants within the selective regions were annotated and selective variants were identified (variants with high consequence impact or missense SNPs, with ΔAF > 0.75). A total of 40 selective variants were identified, including 36 missense SNPs, 1 splice acceptor SNP, 1 splice donor SNP, and 2 frameshift InDels, which were mapped to 25 genes (Additional file 1: Table S8). These variants had very high allele frequencies in the cold group (≥ 0.87) and low frequencies in the hot group (≤ 0.23), while middle frequencies in the warm group (0.52–0.79). These selective variants were validated in the validation population consisting of 119 cattle of seven breeds from cold regions, and their allele frequencies were also found to be very high (≥ 0.83) (Additional file 1: Table S8), further implying they are related to cold adaptation.

### Selective regions and variants identified in the hot group using SNPs and InDels

**Fig. 4:**
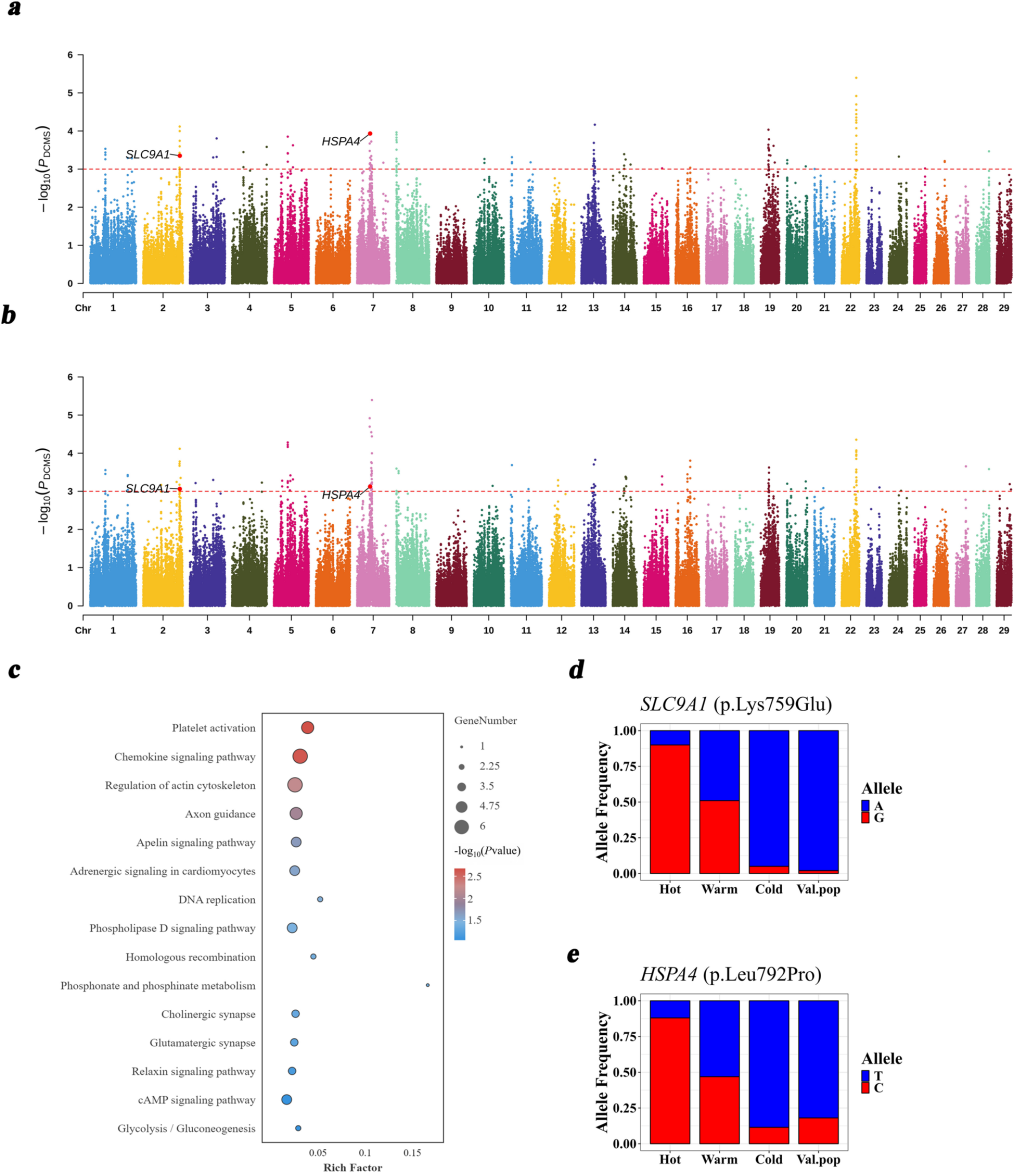
Identification of selective sweeps in the hot group based on SNP and InDel data. **a** and **b** Manhattan plots of the *P*-values (−log_10_(*P*)) (*y*-axis) in sliding 20 kb-windows across all autosomes (*x*-axis) based on SNP data and InDel data. The red dashed line indicates the significance threshold (*P*-value = 0.001). **c** The Sankey-Dot plot of the top 15 KEGG pathways of the selective genes (genes involved in the significant selective regions). **d** and **e** The gene frequencies of the selective variants (missense SNPs) in the *SLC9A1* and *HSPA4* genes in the cold, warm, and hot groups and in the validation population

Using the cold group as the control to identify selective regions in the hot group, 55 selective regions were identified based on the SNP data, encompassing 155 genes (Fig. 4a and Additional file 1: Table S9), and 74 selective regions based on the InDel data, involving 180 genes (Fig. 4b and Additional file 1: Table S10). The correlation coefficient between the *P*-values derived from the SNP and InDel windows was 0.68. A total of 33 selective regions and 63 genes detected based on InDels overlapped with those identified based on SNPs.

KEGG analysis for the 272 genes involved in the selective regions identified 11 significant pathways (Fig. 4c and Additional file 1: Table S11). These pathways were primarily related to the immune system (Platelet activation, Chemokine signaling pathway), signal transduction (Apelin signaling pathway), and the circulatory system (Adrenergic signaling in cardiomyocytes). Using the STRING database, a PPI network consisting of 32 interconnected genes was constructed (Additional file 4: Fig. S3). Among them, the five genes, *HSPA4*, *ATP5F1B*, *SIL1*,* GTPBP4*,* and MON1A*, with the strongest connectivity were identified as hub genes.

The annotation of the SNPs/InDels in the selective regions revealed 51 selective variants, including 49 missense SNPs, 1 splice acceptor SNP and 1 start lost SNP (Additional file 1: Table S12). These variants were localized to 33 genes. In contrast to the selective variants found in the cold group, these variants had high allele frequencies in the hot group (≥ 0.76) and low frequencies in the cold group (≤ 0.12), and again medium frequencies in the warm group (0.27–0.51). In the validation population, frequencies quite similar to those in the cold group were observed (≤ 0.18) (Additional file 1: Table S12), further suggesting these variants are related to hot adaptation.

### Selective SVs

**Fig. 5:**
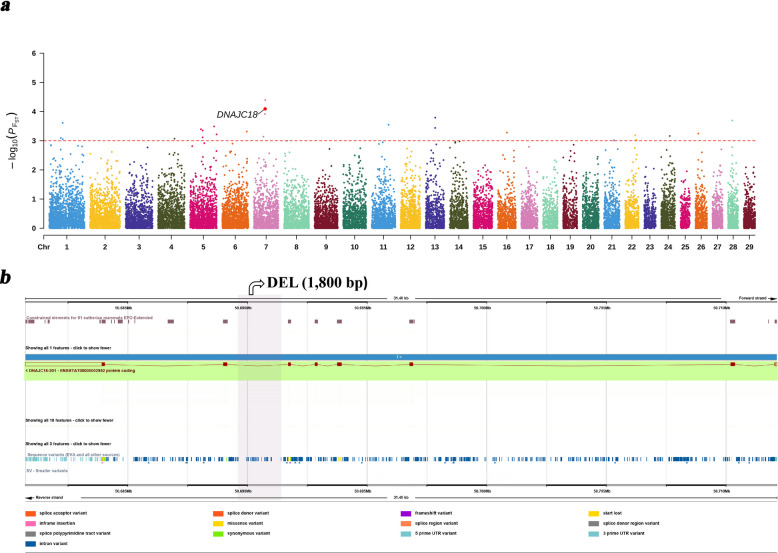
Significantly differentiated SVs between the cold and hot group. **a** Manhattan plot of the *F*_*ST*_ values (*y*-axis) between the cold and the hot group across all autosomes (*x*-axis), the red dashed line represents the significance threshold (*P*-value = 0.001). **b** Gene structure of *DNAJC18* (Chromosome 7: 50,680,729–50,712,129), with the shaded region indicating the location of the 1,800-bp deletion (50,689,611–50,691,410)

A total of 24 selective SVs, i.e., significantly differentiated SVs (*P* < 0.001; corresponding to *F*_*ST*_ > 0.85), including 22 DELs and 2 DUPs, were identified between the cold and hot groups. Most of these SVs are located within intronic regions or intergenic regions. Gene annotation revealed that 14 genes covered or overlapped with some of these SVs (Fig. 5a and Additional file 1: Table S13).

Additionally, five differentiated SVs were found to be located within the selective regions identified based on SNP data (two in the cold group exclusively, one in the hot group exclusively, and two in both groups). Six SVs are located within the selective regions identified based on InDel data (two exclusive to the cold group, two exclusives to the hot group, and two shared by both groups).

### Tissue-specificity of selective genes

Tissue-specific regulation of gene expression plays a critical role in adaptive evolution [77]. Leveraging the CattleGTEx dataset, expression profiles of the selective genes involved in the identified selective regions/SVs were analyzed across 23 distinct tissues, and 83 genes demonstrating high tissue specificity were identified (*tau* value ≥ 0.8; Additional file 1: Table S14).

## Discussion

In this study, population structure and selective sweeps were analyzed using sequence data of 336 cattle representing 21 breeds from three climatic regions: the cold region (6 northern Chinese indigenous breeds and 2 foreign taurine breeds), the hot region (7 southern Chinese indigenous breeds and 3 foreign indicine breeds), and the warm region (3 central Chinese indigenous breeds). Accordingly, these breeds were grouped into three groups, i.e., the cold, hot, and warm groups. The phylogenetic and principal component analyses clearly revealed phylogenetic differentiation between the breeds of the three groups. The admixture analysis further confirmed the distinct clustering of taurine and indicine cattle, with the cold group predominantly consisting of taurine breeds, the hot group mainly comprising indicine breeds, and the warm group displayed an admixture of taurine and indicine lineages (Fig. 1). Furthermore, higher genetic diversity was observed in southern Chinese cattle compared to those in the other two groups, which is consistent with previous studies, and this increased diversity may be attributed to genomic introgression from other *Bos* species [3, 7, 78, 79].

Temperature adaptability is an important aspect of adaptive evolution of farm animals and is subject to long-term natural and artificial selection, resulting in selection signatures in the genome. Identifying these signatures is important for understanding the formation of adaptive traits in different species/breeds and the underlying genetic mechanisms. Considering the extreme difference in regional temperature between the cold and hot breed groups, selective sweep analysis with respect to temperature adaptability was carried out using genome-wide SNPs, InDels, and SVs, respectively. Using SNPs, 86 and 55 selective regions were identified in the cold and hot groups, respectively, covering or overlapping 160 and 155 genes. Using InDels, 103 and 74 selective regions were detected, corresponding to 149 and 180 genes, respectively. Within these selective regions, 40 and 51 selective variants (missense SNPs or variants with high consequence impact, with ΔAF > 0.75) were detected in the cold and hot groups, respectively, which were harbored in 32 and 34 genes, respectively. All these selective variants had clear differences in allele frequency across the three groups corresponding well to the temperature features of the regions where the breeds of the three groups are located, i.e., extremely high or low frequencies in the cold or hot groups and medium frequencies in the warm group. In addition, their frequencies in the validation population containing breeds from cold regions were perfectly consistent with those in the cold group. Using SVs, 24 selective SVs (significantly differentiated SVs between the cold and hot groups with *F*_ST_ > 0.85) were detected. A total of 14 genes harbor or overlap some of these SVs.

Genes related to the selective variants/SVs were analyzed, and several genes, including *KLB*, *HSPA4*, *ECSCR*, *DNAJC18* and *SLC9A1*, were identified as potentially responsible for cold/hot adaptability based on the extreme difference in allele frequency between the cold and hot groups of the selective variants/SVs harbored by these genes, their known biological functions, their roles in the protein–protein interaction network, findings from previous studies on environmental adaptation, and their tissue specificities. Among these, *KLB*, *HSPA4*, and *DNAJC18* have been previously reported to be associated with temperature adaptation in animals, whereas *ECSCR* and *SLC9A1* represent novel candidate genes identified in this study.

*KLB* plays a critical role in regulating glucose oxidation, which is essential for energy acquisition of cells, especially the brain cells and red blood cells, and modulating the thermogenic capacity of adipose tissues [80, 81]. Previous studies have shown that the *KLB* gene dosage is negatively correlated with adiposity, and mice with *KLB* deficiency exhibited reduced thermogenic capacity upon cold exposure [80, 82]. In this study, *KLB* was located within a selective region identified in the cold group based on SNP data (*P* = 9.53 × 10^−4^, Additional file 1: Table S5). Tissue-specific expression analysis revealed that *KLB* is specifically highly expressed in adipose and liver tissues (Fig. 3g, Additional file 1: Table S14). Adipose tissue, particularly brown adipose tissue, plays a critical role in non-shivering thermogenesis, which is essential for heat production in response to cold exposure [83]. This thermogenic process helps maintain body temperature by burning fat to generate heat. Liver also contributes to energy homeostasis during cold stress by regulating processes such as glucose production and lipid metabolism [84]. The high expression of *KLB* in these tissues suggests it may contribute to the regulation of energy balance and the body's adaptive responses to cold environments. Moreover, a missense SNP (p.Ser1012Asn; Fig. 3d) was identified in *KLB*, with its G allele showing very high frequencies in the cold group (0.96) and also in the validation population (0.97), compared to its very low frequency in the hot group (0.09) and medium frequency in the warm group (0.60). The DynaMut2 software [64] was utilized to assess the changes in protein stability and flexibility induced by the p.Ser1012Asn. The analysis indicated that the replacement of serine (Ser) by asparagine (Asn) introduces steric hindrance and alters local flexibility, thereby disrupting the protein's normal conformation and stability (Additional file 5: Fig. S4). Taken together, these findings suggest that *KLB* is a key gene for cold adaptation in cattle.

The HSPA4 protein, a member of the heat shock protein 70 (HSP70) family, the largest and most abundant family of heat shock proteins, enhances cellular thermotolerance by preventing protein denaturation [85]. The *HSPA4* gene was identified as a hub gene in the PPI network of the selective genes in the hot group. It has also been repeatedly identified in several genome-wide selective sweep analyses in indicine cattle as a key candidate gene involved in adaptation to hot environments [86–88]. Consistent with these previous findings, a selective region harboring *HSPA4* was detected in the hot group based on both SNP (*P* = 1.17 × 10^−4^, Additional file 1: Table S9) and InDel (*P* = 1.89 × 10^−4^, Additional file 1: Table S10) data. A missense variant in *HSPA4* (p.Leu792Pro; Fig. 4e) was identified as a selective variant, with its C allele exhibiting a clear decline in frequency along with the temperature gradient: 0.88 in the hot group, 0.47 in the warm group, and 0.11 in the cold group (0.18 in the validation population) (Additional file 1: Table S12). The replacement of the flexible leucine (Leu) residue with proline (Pro), an amino acid with a rigid cyclic side chain, imposes significant conformational constraints on the protein backbone, thereby reducing local flexibility. The introduction of proline facilitates the formation of new stabilizing atomic interactions (depicted as red dashed lines), which collectively contribute to the overall stabilization of the protein structure (Additional file 6: Fig. S5). This enhanced structural stability aligns with its known function as a heat shock protein, suggesting that *HSPA4* is likely to improve cellular thermotolerance and support adaptation to high-temperature environments.

DNAJC18 is a member of the heat shock protein family HSP40. The *DNAJC18* gene has been previously reported to be associated with heat stress in East African Shorthorn Zebu cattle [89] and Ethiopian indigenous sheep [90], as well as with cold adaptation in Chinese indigenous cattle [91]. Consistent with these findings, *DNAJC18* was observed to be located within a strong selective region identified based on both SNP data (*P* = 1.89 × 10^−4^, Additional file 1: Table S5) and InDel data (*P* = 6.84 × 10^−5^, Additional file 1: Table S6) in the cold group. It was identified as a hub gene in the PPI network of the selective gens in the cold group. Notably, no selective SNPs or InDels were detected within this gene. Instead, a selective SV (1,800-bp deletion, *F*_*ST*_ = 0.92; Fig. 5b) was identified in the intronic region of *DNAJC18*. This SV was previously reported by Hu et al. [60] in a comparative analysis of copy number variation between *Bos taurus* and *Bos indicus*. In line with their results, this 1,800-bp sequence was observed to be predominantly present in the cold group (*Bos taurus*), while generally absent in the hot group (*Bos indicus*), indicating strong divergent selection. Introns are increasingly recognized as important reservoirs of *cis*-regulatory elements, with a subset of intronic sequences playing key roles in transcriptional regulation by recruiting transcription factors, modulating alternative splicing, and reshaping higher-order chromatin architecture [25, 92]. It is therefore plausible that loss of this 1,800-bp intronic segment in *Bos indicus* alters the transcriptional regulation of *DNAJC18*, potentially conferring a selective advantage under hot environmental conditions. Conversely, retention of this segment in *Bos taurus* may help maintain the *DNAJC18* expression pattern that is more favorable for coping with cold climates, which is consistent with the known role of *DNAJC18* in protein quality control and protection against proteotoxic stress [93]. Future functional studies dissecting the impact of this SV on *DNAJC18*’s transcriptional dynamics and the activity of the heat shock protein will provide important mechanistic evidence for how non-coding structural variants contribute to adaptive phenotypes in livestock.

Previous studies have shown that *ECSCR* is highly expressed in both white and brown adipose tissues in mice, where it regulates energy metabolism and glucose homeostasis through its influence on endothelial cell function [94]. These functions are supposed to be highly related to the adaptation to cold environments of animals [95, 96]. In this study, *ECSCR* was identified in a strong selective region in the cold-environment population based on both SNP data (*P* = 1.89 × 10^−4^, Additional file 1: Table S5) and InDel data (*P* = 6.84 × 10^−5^, Additional file 1: Table S6). It was also a hub gene in the PPI network of the selective genes in the cold group. Furthermore, a missense SNP (p.Met177Val; Fig. 3e) and a frameshift InDel (p.Gly99fs; c.295_296insCTTCCACTCAGAG; Fig. 3f) were identified in *ECSCR*, both of which exhibited a clear gradient in allele frequencies across the cold, warm, and hot groups (Additional file 1: Table S8), with extreme differences between the cold and hot groups (ΔAF = 0.93 and 0.92, respectively) and very high frequences in the validation population (0.97), suggesting their potential role in cold adaptation. The DynaMut2 tool [64] revealed that the p.Met177Val mutation destabilizes the protein by reducing side chain volume, eliminating the sulfur atom, and altering local flexibility (Additional file 7: Fig. S6). In addition, SWISS-MODEL [97] was used to predict the protein structures of both the wild-type and mutant forms of the InDel p.Gly99fs, revealing that this InDel causes local folding disruption in the protein structure and extension of the polypeptide chain (Additional file 8: Fig. S7). This structural change may affect the protein's function and potentially alter its role in regulating vascular and adipose tissue functions. Overall, these findings support the notion that the *ECSCR* gene plays a crucial role in temperature adaptation by regulating the functions of vascular and adipose tissues.

The *SLC9A1* gene encodes the Na⁺/H⁺ exchanger NHE1, a ubiquitously expressed integral plasma membrane protein that finely regulates intracellular pH by exchanging one intracellular H⁺ for one extracellular Na⁺, thereby playing a pivotal role in maintaining acid–base homeostasis in mammalian cells [98]. In this study, *SLC9A1* was found to be located within a selective region identified in the hot group using both SNP data (*P* = 4.46 × 10^−4^, Additional file 1: Table S9) and InDel data (*P* = 8.65 × 10^−4^, Additional file 1: Table S10). KEGG pathway analysis indicated that *SLC9A1* is primarily involved in the Apelin signaling pathway and adrenergic signaling in cardiomyocytes, whereby the Apelin signaling pathway serves as an important regulator of cardiac and vascular function and contributes to adaptation to physiological stress and disease [99], while adrenergic signaling in cardiomyocytes is critical for the regulation of cardiac function, particularly under high-temperature stress [100]. Additionally, a missense SNP (p.Lys759Glu; Fig. 4d) was identified in *SLC9A1*, with its G allele showing a distribution pattern across the breeds of different regions that was perfectly consistent with the temperature features of these regions (Additional file 1: Table S12). This mutation replaces the positively charged lysine (Lys) with the negatively charged glutamic acid (Glu), leading to electrostatic repulsion and steric clashes, which could destabilize the protein structure (Additional file 9: Fig. S8). These findings suggest that this mutation is likely responsible for adaptation to heat stress by affecting protein stability and Na⁺/H⁺ exchange function.

SNPs, InDels, and SVs represent three major classes of genomic variants, among which SNPs are currently the most extensively utilized variant. In recent years, ‌ population genetic studies have increasingly incorporated InDel and SV data into analytical frameworks to corroborate and complement findings derived from SNPs alone [20, 101–104]. For example, Zhao et al. [104] conducted a genome-wide association study for dairy traits in Holstein cattle using both InDel and SNP markers, finding that although most significant InDels were captured by nearby SNPs due to strong linkage disequilibrium, nine InDels showed independent associations with milk production traits, thus providing an important complement to SNP-based studies. Qiu et al. [103] constructed a comprehensive SNP and SV dataset from 418 globally sampled pigs, revealing the limitations of SNP-based analyses for capturing SV genetic effects, and, through joint SNP-SV analysis, identified 3,558 bidirectionally introgressed genomic segments and 30 SVs, delineating the bidirectional introgression landscape between Chinese and European pig populations. In this study, the DCMS values for the same windows calculated from SNP and InDel data showed limited concordance, with correlation coefficients of 0.57 and 0.68 in the cold and hot groups, respectively. Moreover, the overlap between selective regions detected from SNPs and InDels was limited, with 15 regions overlapping in the cold group (Additional file 1: Table S6) and 33 in the hot group (Additional file 1: Table S10). Among the 24 selective SVs, only eight overlapped with the selective regions identified based on SNPs or InDels (Additional file 1: Table S13). When focusing on selective variants, two frameshift InDels were identified, which may alter the coding sequence of the protein, resulting in significant structural changes that typically lead to gene function loss, protein instability, or functional alterations. It has been reported that gene silencing induced by a frameshift mutation is one of the key mechanisms of adaptive evolution [105–107]. Even when the same selective gene was identified using different types of variants, they may affect the gene’s function through different mechanisms, thereby providing multilayered support for the involvement of the gene in adaptive processes. For example, a selective missense SNP and a selective frameshift InDel were identified for *ECSCR* (Additional file 1: Table S8), which may jointly modulate the structures and functions of the proteins encoded by this gene. On the other hand, there were genes for which only InDel or SV was detected as putative selective variants. In particular, most of the selective SV was not overlapping with the selective regions identified based on SNP or InDel, indicating the potentially important contribution of SV to temperature adaptation. Therefore, incorporating InDel and SV data alongside SNPs provides a powerful complement, enabling a more comprehensive characterization of the genetic basis of environmental adaptation.

By integrating different genomic variant data (SNPs, InDels, and SVs), selective genes and genomic variants associated with temperature adaptation (cold and hot tolerance) were identified, providing insights into temperature adaptation in cattle. However, this study has several limitations. First, a taurine-based cattle reference genome was used, which may introduce bias when detecting and interpreting indicine-specific variants. Second, although the sequencing depth for SV detection was relatively high (> 30 ×), SV discovery based on short-read sequencing still cannot fully avoid false positives and false negatives. Third, the analyses mainly focused on selective variants, which were either missense SNPs or variants with high impact on protein sequence, while the potential regulatory roles of other variants were not evaluated. In the future, the application of cattle pangenomes and long-read sequencing, together with multi-omics integration and functional validation, is expected to enable a more refined dissection of the genetic basis of thermal adaptation and provide more robust molecular targets for breeding hot- and cold-tolerant cattle.

## Conclusions

Using whole-genome sequence data from 336 individuals representing 21 cattle breeds, we revealed the pronounced genetic differentiation among three climate-defined breed groups. By integrating genome-wide SNP, InDel, and SV data, we identified a series of candidate genes and variants associated with temperature adaptation. Some of the genes, such as *KLB*, *HSPA4*, and *DNAJC18*, have been previously reported to be implicated in thermal adaptation in animals, whereas some, such as *ECSCR* and *SLC9A1,* are novel identified in this study. Moreover, our results show that jointly utilizing InDel and SV information together with SNP data enables a more comprehensive and nuanced dissection of the genetic basis of environmental adaptation and provides a robust framework for incorporating diverse classes of genomic variants into adaptive evolution studies in other species. Overall, these findings deepen our understanding of the mechanisms underlying thermal adaptation in cattle and provide an important molecular foundation for the development of climate-resilient cattle populations.

## Supplementary Information

Additional file 1: Table S1. Sequencing information of the 338 individuals involved in this study. Table S2. Numbers of SNPs, InDels, and SVs and their densities on each chromosome. Table S3. Distance matrix among the 21 cattle breeds based on SNPs. Table S4. Nucleotide diversities in the 21 cattle breeds. Table S5. Significant selective regions identified in the cold groups based on SNPs with the hot group being used as control. Table S6. Significant selective regions identified in the cold groups based on InDels with the hot group being used as control. Table S7. Significant KEGG pathways of genes involved in the selective regions identified in the cold group. Table S8. Selective variants identified in the cold group. Table S9. Significant selective regions identified in the hot groups based on SNPs with the cold group being used as control. Table S10. Significant selective regions identified in the hot group based on InDels with the cold group being used as control. Table S11. Significant KEGG pathways of genes involved in the selective regions identified in the hot group. Table S12. Selective variants identified in the hot group. Table S13. Significantly differentiated SVs between the cold and hot groups. Table S14. Tissue specificity indices and expression profiles) of the selective genes in different cattle tissues.

Additional file 2: Fig. S1. Population-level phylogeny inferred using the maximum likelihood approach implemented in TreeMix.

Additional file 3: Fig. S2. Protein–protein interaction network of selective genes in the cold group.

Additional file 4: Fig. S3. Protein–protein interaction network of selective genes in the hot group.

Additional file 5: Fig. S4. Local protein structures of the wild-type and mutant-type of the KLB protein due to the p.Ser1012Asn mutation.

Additional file 6: Fig. S5. Local protein structures of the wild-type and mutant-type of *HSPA4* due to the p.Leu792Pro mutation.

Additional file 7: Fig. S6. Local protein structures of the wild-type and mutant-type of *ECSCR* due to the p.Met177Val mutation.

Additional file 8: Fig. S7. Protein structures of the wild-type and mutant-type of *ECSCR* due to the p.Gly99fs mutation.

Additional file 9: Fig. S8. Local protein structures of the wild-type and mutant-type of *SLC9A1* due to the p.Lys759Glu mutation.

## Data Availability

The raw sequence data for a subset of animals (*n* = 101) used in this study were downloaded from NCBI (see Table S1). The sequence data for the remaining animals (*n* = 235) are available upon request for research purposes.

## Abbreviations

DCMS: De-correlated composite of multiple signals
*F*_*ST*_: Fixation statistic
InDel: Insertion/deletion
KEGG: Kyoto Encyclopedia of Gene and Genome
LD: Linkage disequilibrium
MAF: Minor allele frequency
NCBI: National Center for Biotechnology Information
NJ: Neighbor-Joining
PCA: Principal component analysis
PPI: Protein–protein interaction
SVs: Structural variations
WGS: Whole genome sequencing
