# Supplementary figures and images for: Genome-wide variation landscape reveals temperature adaptation in Chinese indigenous cattle
Source: J Anim Sci Biotechnol. 2026 Jul 1;17:135. doi: 10.1186/s40104-026-01451-6 (PMC13321616; doi:10.1186/s40104-026-01451-6)

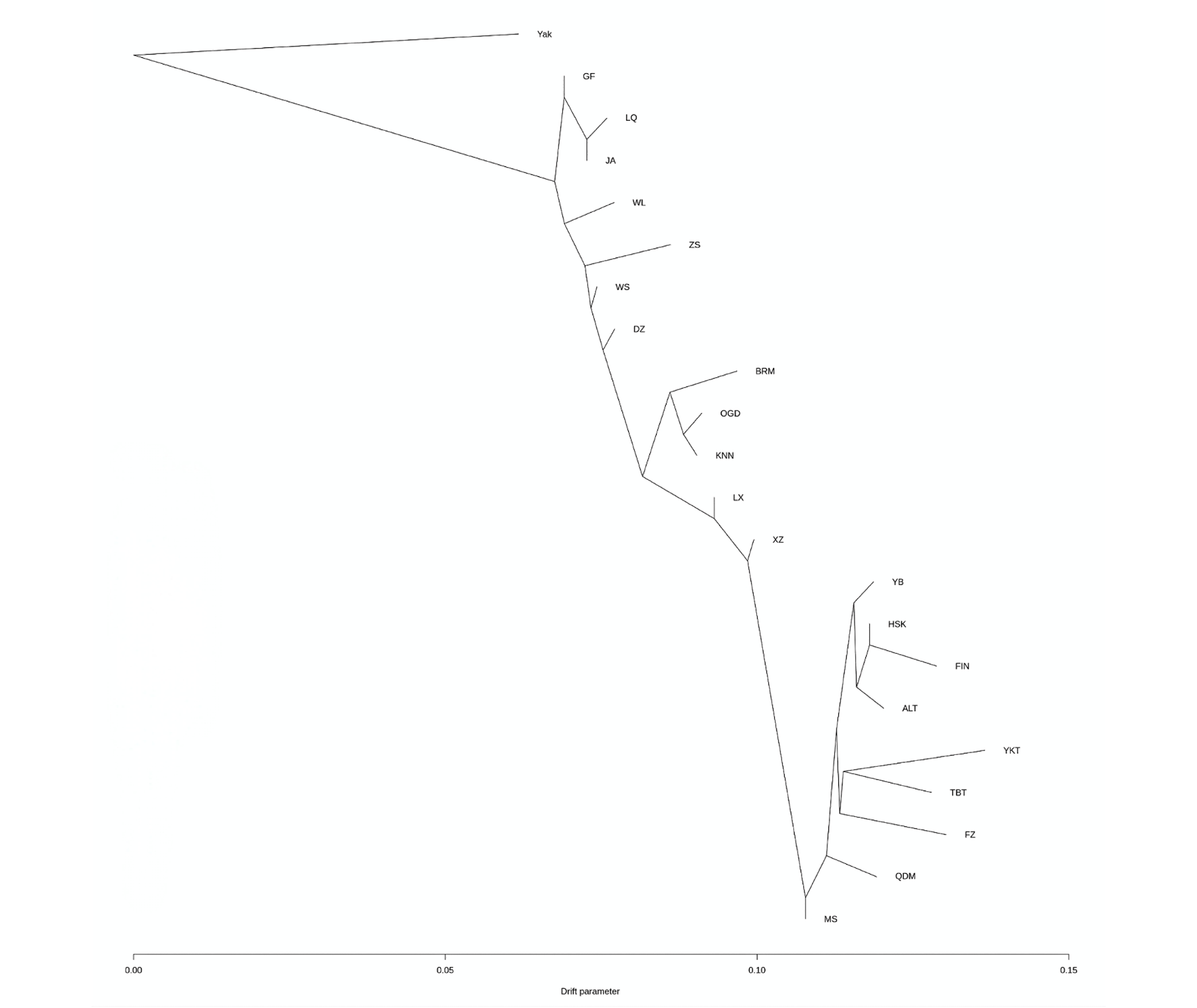

Supplement: Supplementary file 2 — Additional file 2: Fig. S1. Population-level phylogeny inferred using the maximum likelihood approach implemented in TreeMix. [file 40104_2026_1451_MOESM2_ESM.tif]

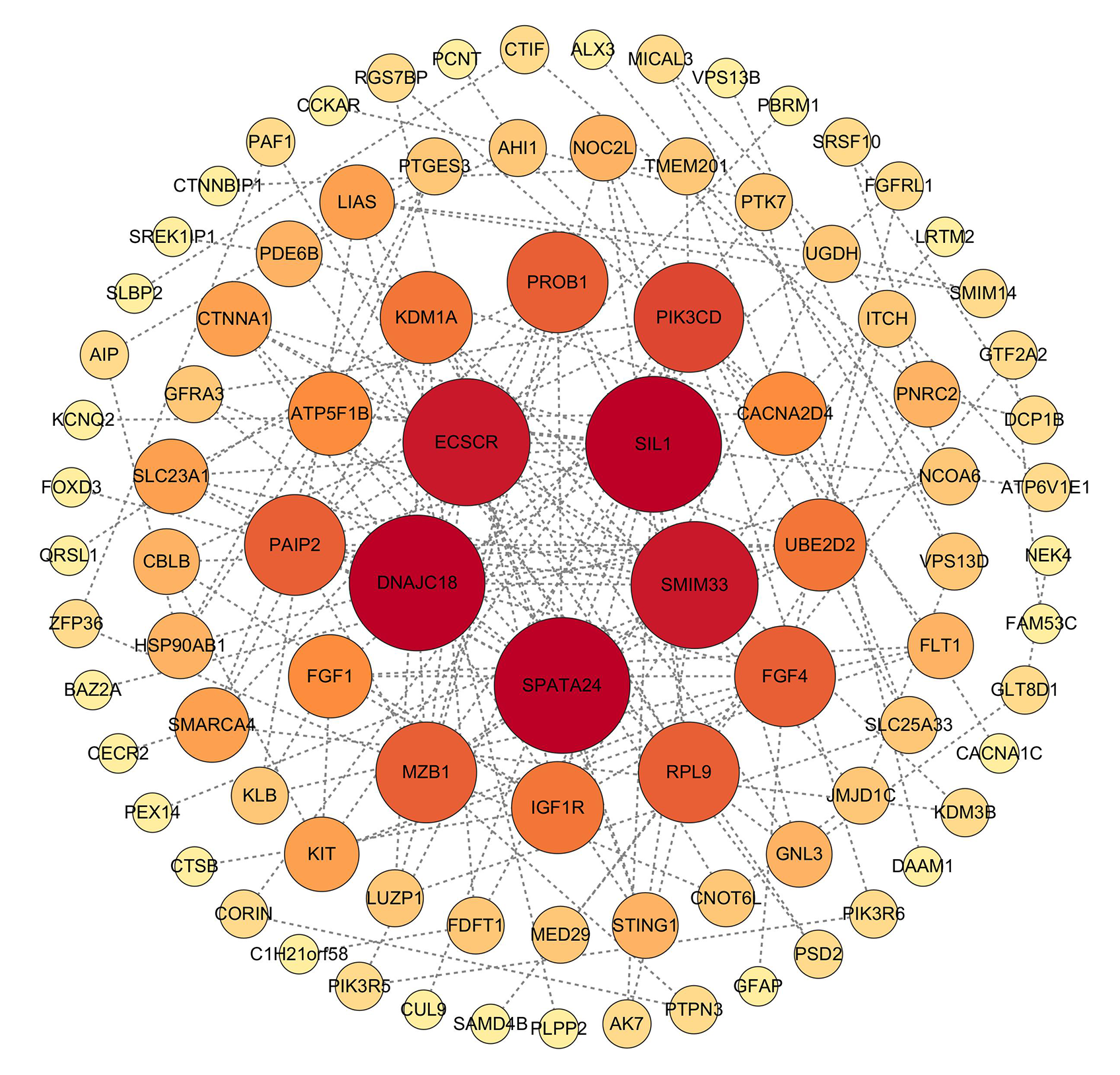

Supplement: Supplementary file 3 — Additional file 3: Fig. S2. Protein–protein interaction network of selective genes in the cold group. [file 40104_2026_1451_MOESM3_ESM.tif]

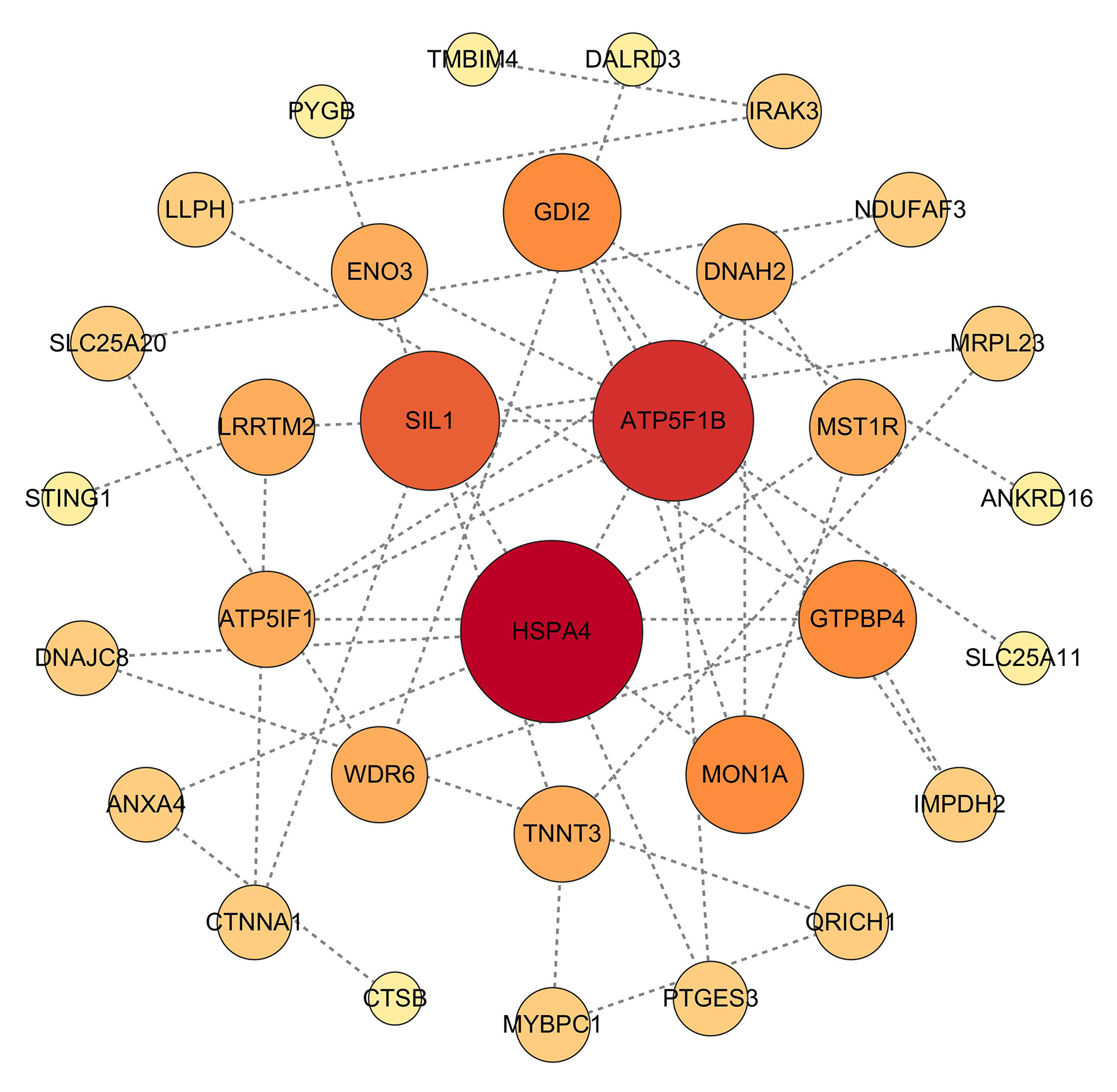

Supplement: Supplementary file 4 — Additional file 4: Fig. S3. Protein–protein interaction network of selective genes in the hot group. [file 40104_2026_1451_MOESM4_ESM.tif]

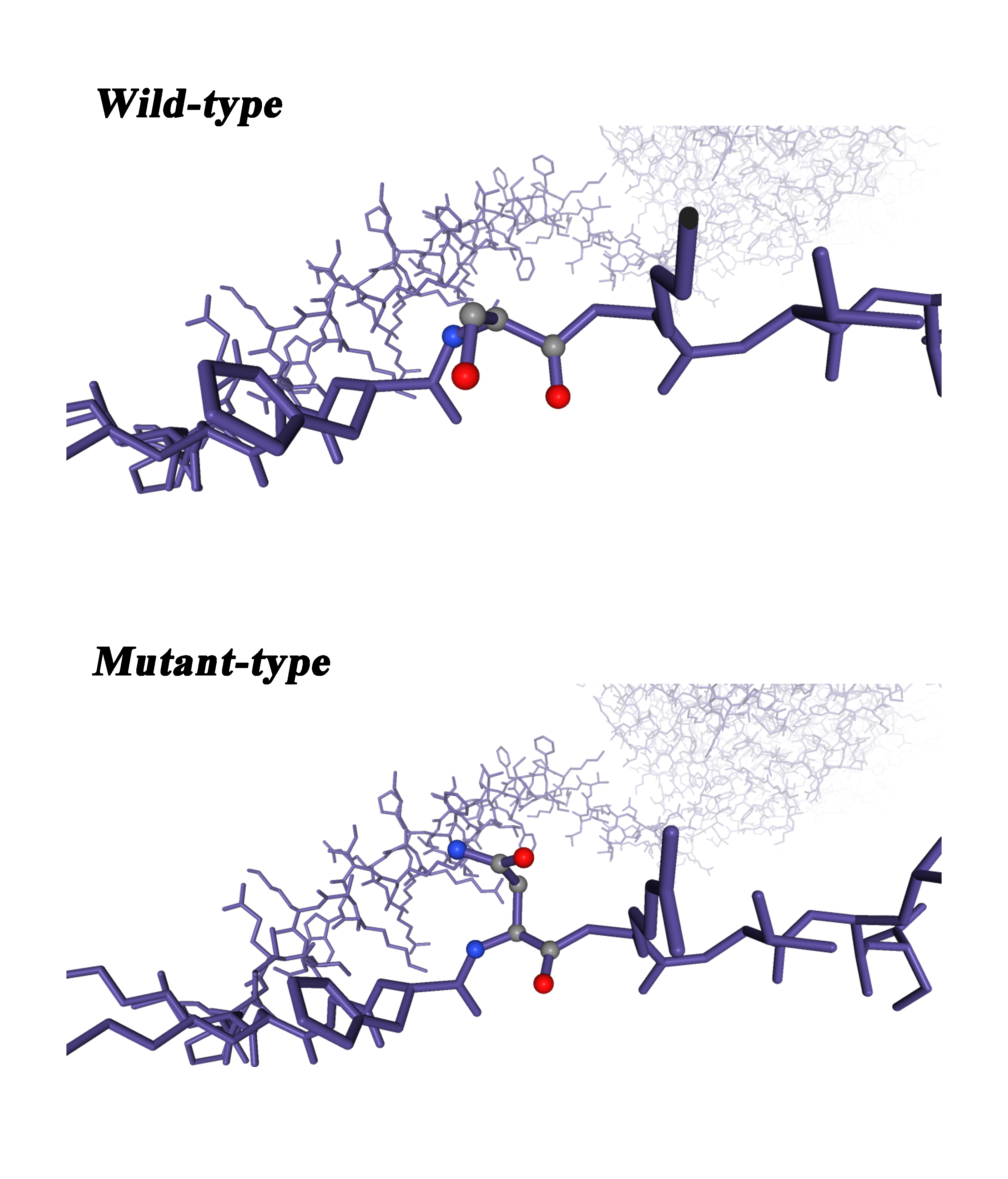

Supplement: Supplementary file 5 — Additional file 5: Fig. S4. Local protein structures of the wild-type and mutant-type of the KLB protein due to the p.Ser1012Asn mutation. [file 40104_2026_1451_MOESM5_ESM.tif]

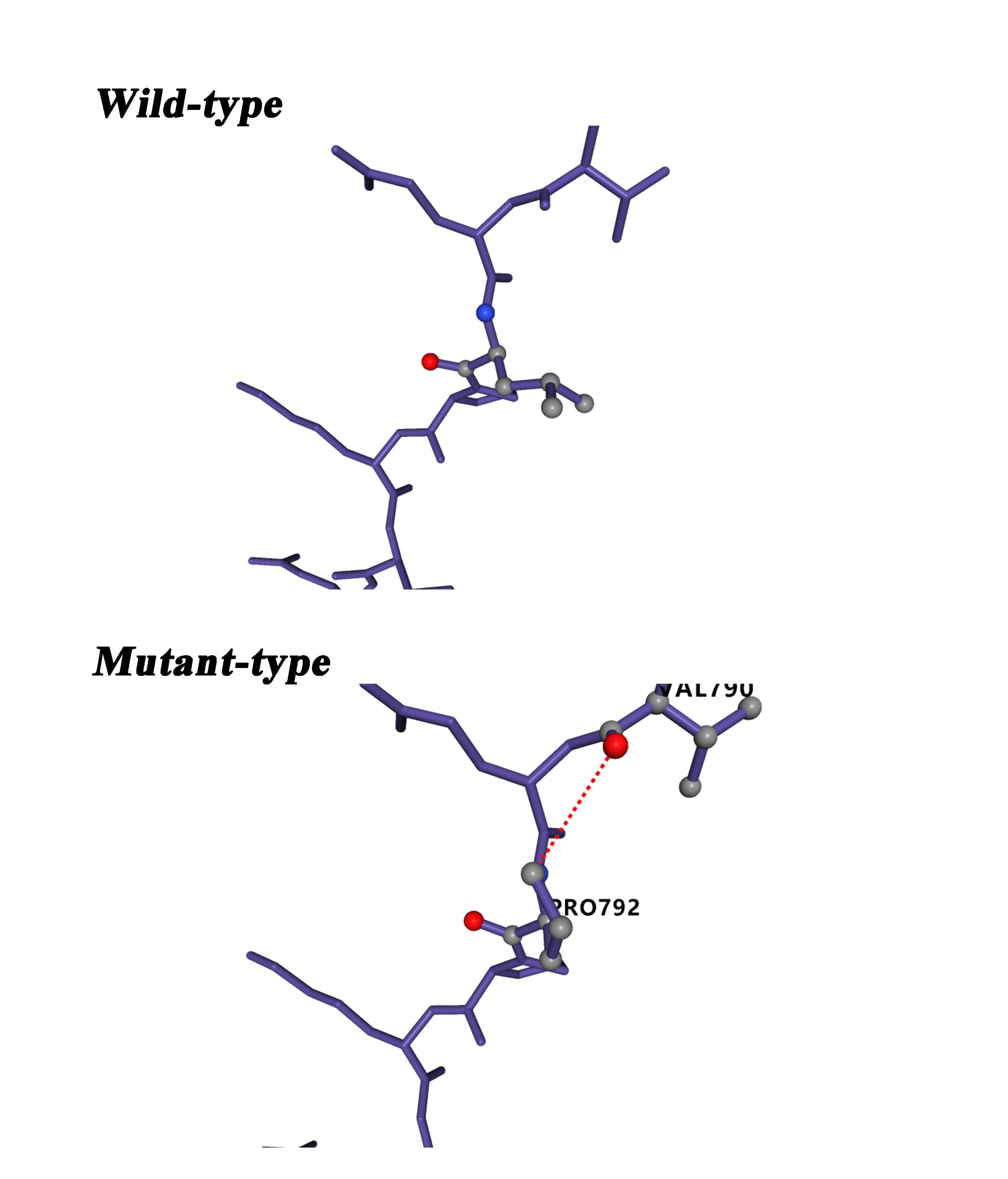

Supplement: Supplementary file 6 — Additional file 6: Fig. S5. Local protein structures of the wild-type and mutant-type of HSPA4 due to the p.Leu792Pro mutation. [file 40104_2026_1451_MOESM6_ESM.tif]

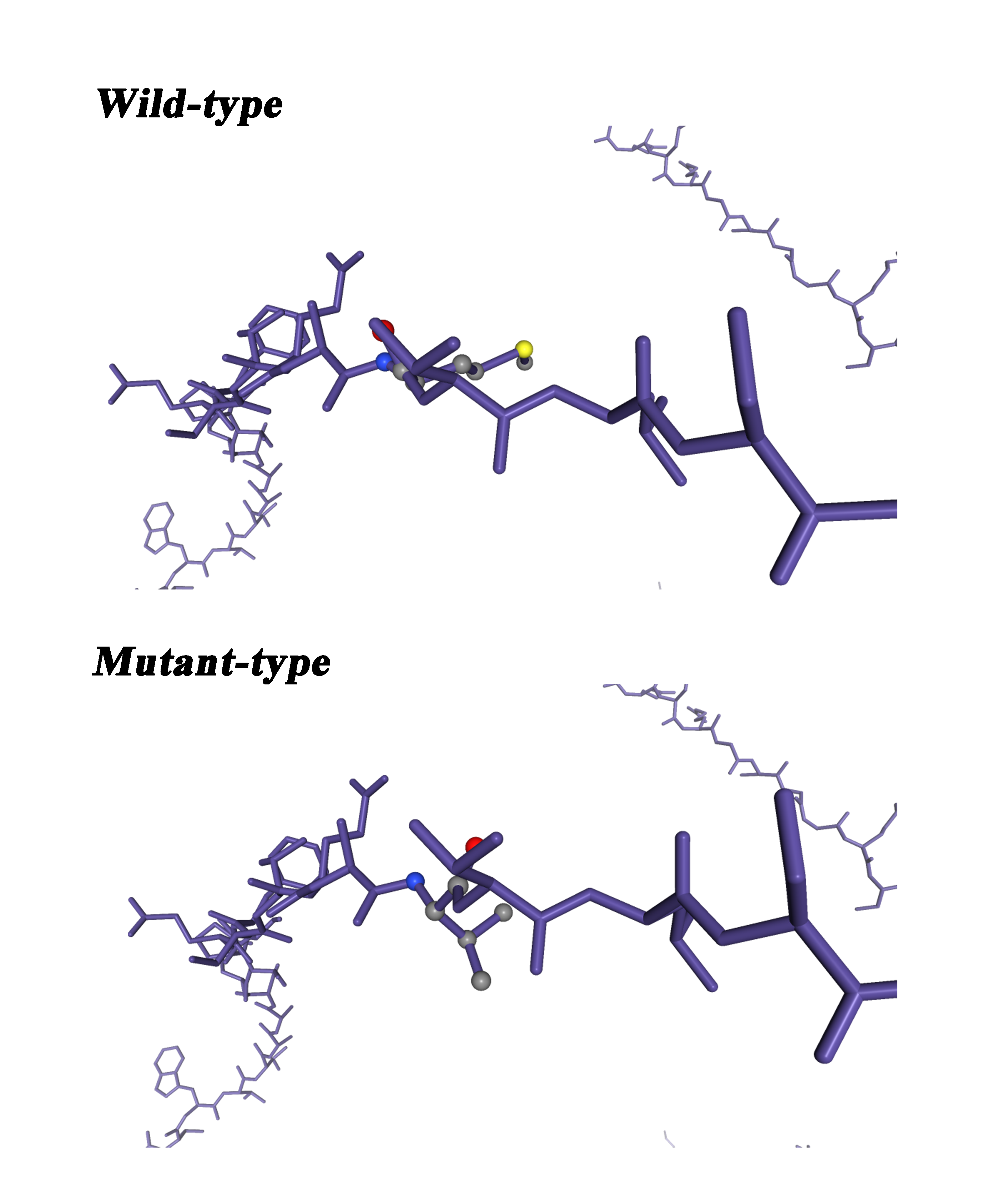

Supplement: Supplementary file 7 — Additional file 7: Fig. S6. Local protein structures of the wild-type and mutant-type of ECSCR due to the p.Met177Val mutation. [file 40104_2026_1451_MOESM7_ESM.tif]

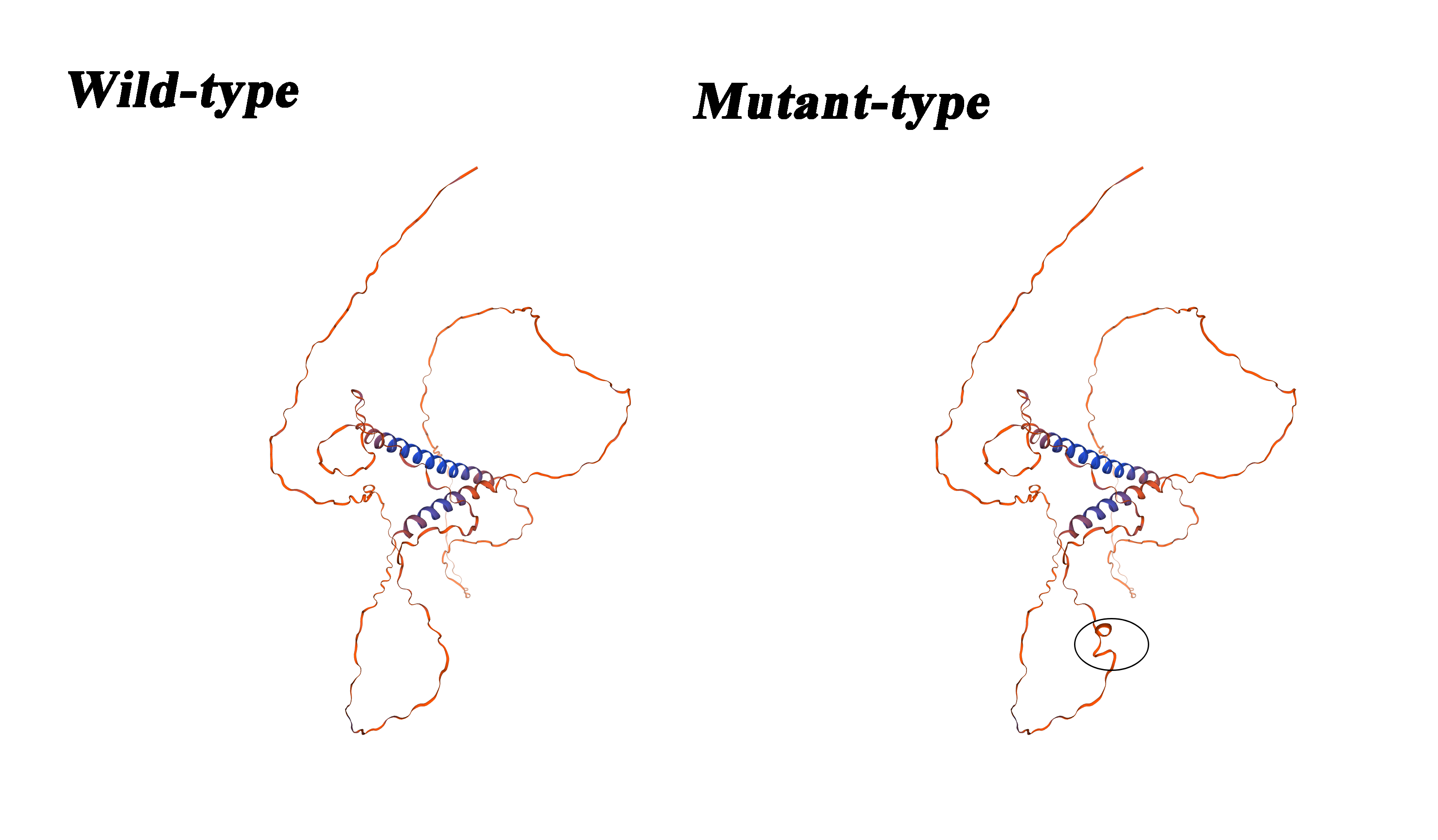

Supplement: Supplementary file 8 — Additional file 8: Fig. S7. Protein structures of the wild-type and mutant-type of ECSCR due to the p.Gly99fs mutation. [file 40104_2026_1451_MOESM8_ESM.tif]

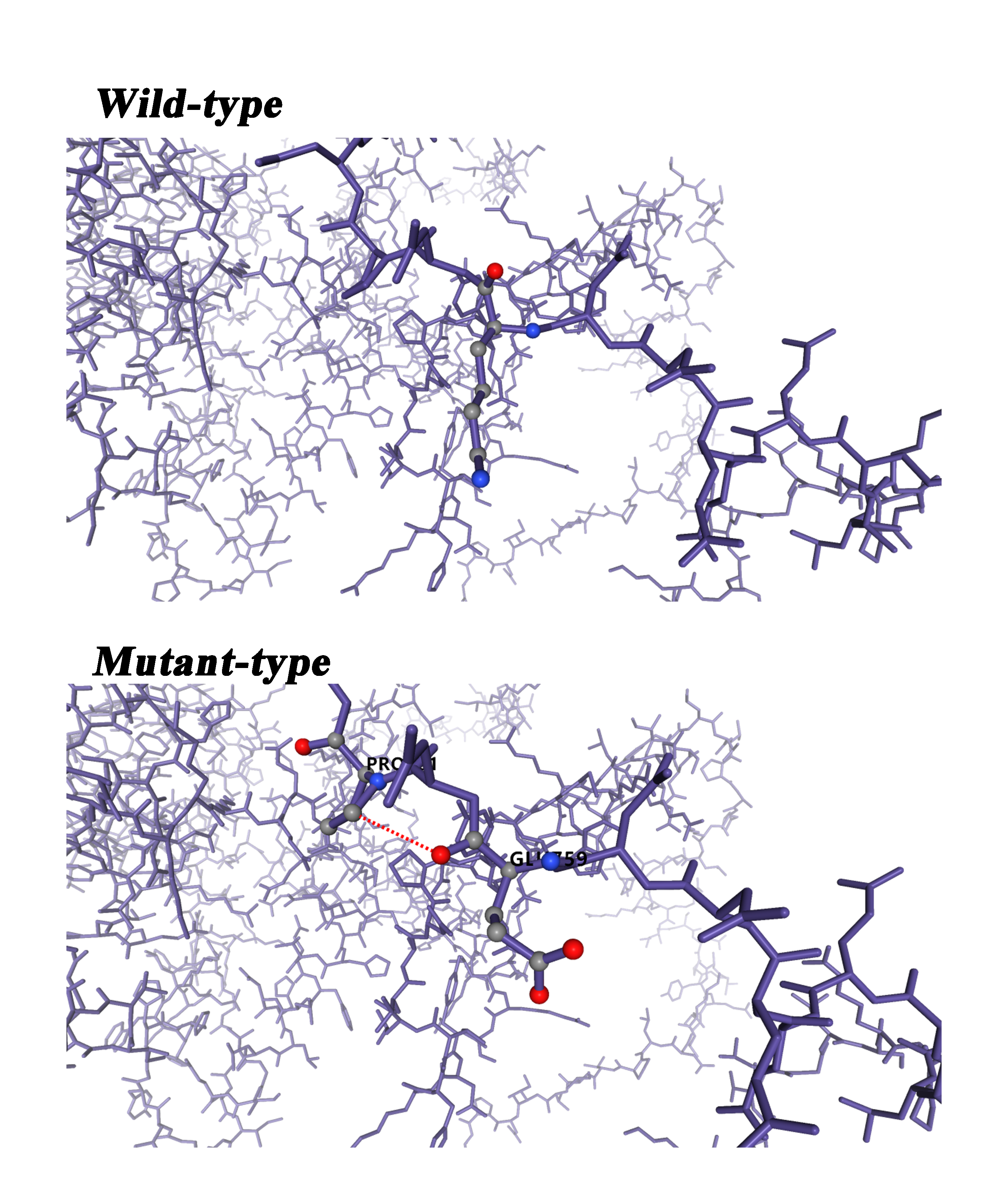

Supplement: Supplementary file 9 — Additional file 9: Fig. S8. Local protein structures of the wild-type and mutant-type of SLC9A1 due to the p.Lys759Glu mutation. [file 40104_2026_1451_MOESM9_ESM.tif]
